# Impact of malathion toxicity on the oxidative stress parameters of the black soldier fly *Hermetia illucens* (Linnaeus, 1758) (Diptera: Stratiomyidae)

**DOI:** 10.1038/s41598-022-08564-8

**Published:** 2022-03-17

**Authors:** Eman Alaaeldin Abdelfattah, Ghada M. El-Bassiony

**Affiliations:** Department of Entomology, Faculty of Science, Cairo University, El-Nahda Square, Giza, Cairo, 12613 Egypt

**Keywords:** Biochemistry, Biological techniques, Biotechnology, Immunology, Environmental sciences

## Abstract

The black soldier fly larvae (BSFL) may serve as a promising tool in the animals feed production industry. The input organic wastes may be contaminated by insecticides that affect both the insect’s mass rearing, and the animals feed process. Therefore, in the current study the assessment of oxidative stress parameters of the black soldier fly (BSF) were investigated to quantify the deleterious effect of malathion-contaminated kitchen waste (1:1 vegetable: fruit waste) container on the insect. The different developmental stages of insect (adult and larva) were exposed to different concentrations (0, 0.005, 0.01, 0.015, and 0.02 mg/mL) of malathion. The results showed that the mean value of the reactive oxygen species (ROS), which included hydrogen peroxide (H2O2) and superoxide anion radicals (O2^•-^) concentrations were lower in larval stage than in adults, in all treated groups (0, 0.005, 0.01, 0.015, and 0.02 mg/mL malathion concentration). Also, the protein carbonyls amount and lipid peroxides levels were decreased in the 0.02 mg/mL Malathion compared to the control values. However, the cluster analysis revealed slight dissimilar patterns for control insects and the highest malathion concentration (0.02 mg/ml). These stage-related differences could occur from the different growth dynamic functions of larvae and adults. The larvae were distinguished by robust growth, and significant oxygen consumption. The results verified that oxidative stress parameters, especially protein carbonyls and α, α-diphenyl-β-picrylhydrazyl (DPPH) were promising, cheap, quick and cost-effective applications for determining the macromolecules damage, and antioxidant ability of *H. illucens* enclosed with malathion exposure. These findings described that malathion application induces macromolecules damage mediated through oxidative stress injury.

## Introduction

The organic waste contamination poses a grave threat on the environment^1^. This required the contaminants and pollutants to be assessed; to protect the environment and the living organism's health^2^. Food processing could be affected by environmental pollutants. Briefly, the whole life cycle of food industry, from cradle to grave stages, may lead to maximizing the hazards of organic waste recycling^3^. Moreover, the food industry cradle stage, which include crop production, may include the pesticides contamination. The pesticides are used to avoid the negative impact of different pests on crop productivity^4^. Nowadays, the production and consumption of pesticides have been applied for agricultural and non-agricultural practices, until they reached more than two million tons’ consumption/year^5^. The pesticide fate, transport, and dispersion may have a harmful effect on the natural ecosystems including biodiversity loss; impact on non-target species; or even adverse effect on air, soil, and water quality^6^. Therefore, the pesticide consumption was considered as an interfering agent to the environment quality and various vital processes such as photosynthesis, biosynthesis reactions and microbes' molecular composition^7^. organophosphate (OP) insecticides were famous due to their ability to accumulate with low toxicity and persistence rate^8^. The action mechanism of OP insecticides depends on the degradation and interaction processes. The degradation process includes breaking down of malathion into malaxon. Then, it interacts with the active site of acetylcholinesterase leading to inhibition of acetylcholine hydrolysis and paralysis. In addition, the toxicity of pesticides, especially malathion, depended on oxygen free radicals' induction^9,10^.

The grave stage of the food industry was concerned with the recycling of organic wastes which may contaminated with pesticides^11^. Insects are widespread and are characterized by their sensitivity to environmental changes and their ability to be used in the biomonitoring and bioremediation programs^12,13^. In addition, insect’s oxidative stress parameters could be used to quantify the pollutants effect, like malathion exposure level, on the living organisms. Nowadays, BSF was considered as a biotechnology tool, due to its ecofriendly behavior; it was known for its capability to ensure the circular economy concept^14^, and its ability to solve the contamination problems especially those dealt with agricultural and organic waste management^13,15,16^.

Generally, the oxidative stress of the contaminated ecosystem could occur internally and externally, as a result of imbalance status between ROS and antioxidants^17,18^. The ROS included O_2_^•-^, H_2_O_2_, singlet oxygen, peroxyl radical, nitric oxide and hydroxyl radical (^•^OH)^19^. When the ROS levels exceeded the antioxidant’s levels, they led to macromolecules damage in the form of protein carbonyls, enzyme inactivation, lipid peroxides, and genotoxicity^13,20,21^. Antioxidants included non-enzymatic antioxidants (such as reduced glutathione (GSH), α-tocopherol, ascorbic acid, and β-carotene), and enzymatic antioxidants (such as superoxide dismutase (SOD), catalase (CAT), peroxidase (Px), polyphenol oxidase (PPO), ascorbate peroxidase (APOx), and acetylcholine esterase (AChE))^22^. In addition, the antioxidant non-enzymatic activity can be detected by DPPH assay, in which antioxidants can inhibit the lipid oxidation. So, the scavenging rate of DPPH radical can determine free-radical scavenging capacity ^23^. The potential usage of oxidative stress parameters, to assess the impacts of malathion on insect bioreactor, especially BSF fed on organic waste, wasn’t studied before^24^.

This work aimed to assess the impact of malathion-contaminated organic waste (fruits and vegetables) on the oxidative stress parameters of BSF. We measured ROS (H_2_O_2_ and O_2_^•-^) concentration, macromolecules damage (protein carbonyls and lipid peroxides), enzymatic antioxidant response (SOD, CAT, and PPO) and non-enzymatic antioxidants (DPPH and GSH) in the midgut homogenates of 5th larval instar (MHL) and male adult (MHA) of BSF, which were exposed to different malathion concentration (0, 0.005, 0.01, 0.015, and 0.02 mg/ mL).

## Results

### The concentration of ROS

**Figure 1:**
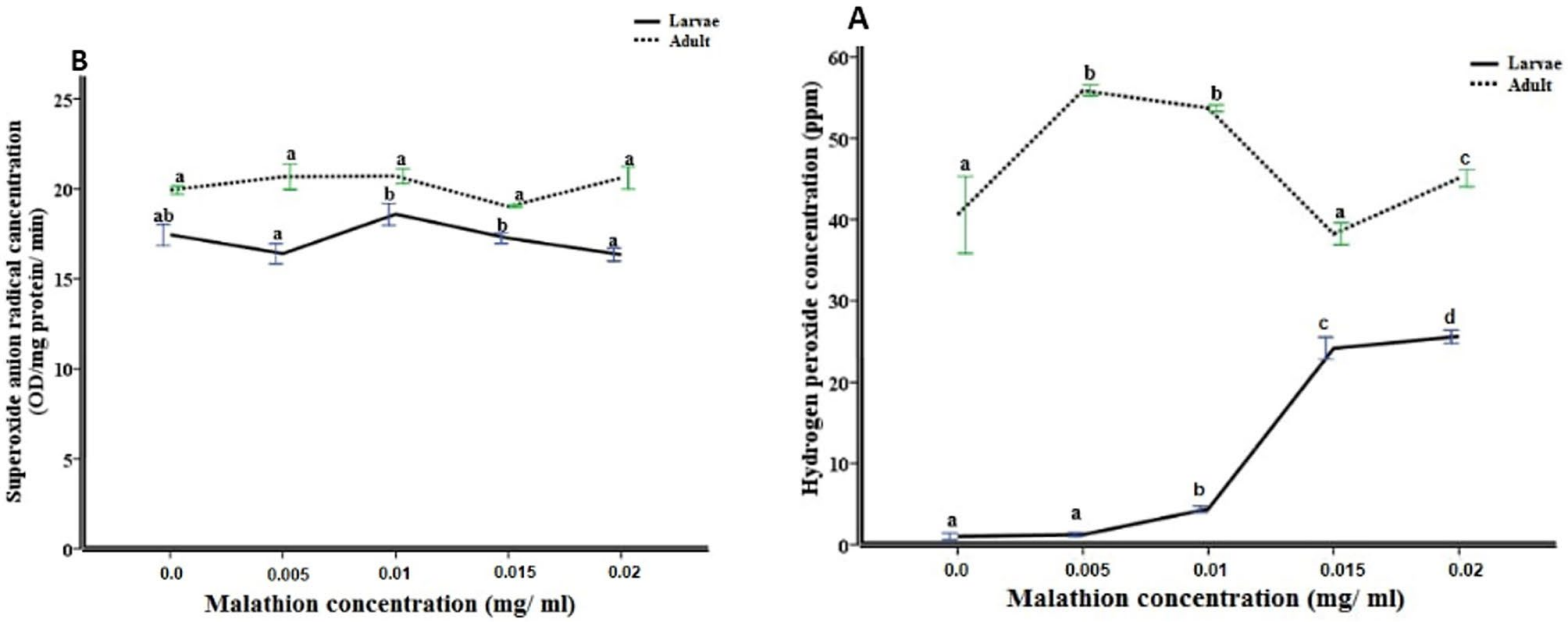
Reactive oxygen species (ROS) concentration, that expressed as hydrogen peroxide (H_2_O_2_) concentration (**A**), and superoxide anion (O_2_^•-^) concentration (**B**). The data were expressed as mean ± SE. The concentrations were obtained from gut homogenates of 5th instar larvae and males of *Hermetia illucens*, which their food containers were exposure to different concentrations of malathion (0, 0.005, 0.01, 0.015, 0.02 mg/ml). Mean values marked with the different lowercase letters were significantly different among malathion concentration assessment (ANOVA test *Tukey's-b*, Post Hoc test, *p* < 0.05).

The concentration of H_2_O_2_ and O_2_^•-^ in MHL and MHA, which were exposed to different malathion concentration; were shown in Fig. 1. The ANOVA test, *Tukey's-b*, Post Hoc test showed that the results of H_2_O_2_ included SS = 1878.6 and 735.7, MS = 469.6 and 183.9, F = 1075.6 and 46.9, df = 4; and *p* value < 0.001 in larval and adult stages, respectively. Also, the results of O_2_^•-^, in both larval and adult stages included SS = 10.13 and 6.35, MS = 2.5 and 1.6, F = 13.5 and 9.4, df = 4; and *p* value < 0.001, respectively. The mean value of both H_2_O_2_ and O_2_^•-^ were lower in larvae than adults at all experimental concentration of malathion. The H_2_O_2_ concentration has direct correlation with malathion concentration at the larval stage of *H. illucens* (Fig. 1A); however, the highest H_2_O_2_ concentration was recorded at the malathion concentration 0.005 and 0.015 mg/ml in the adult males and larva, respectively (Fig. 1A). The O_2_^•-^ production rate showed non-significant elevations/ depressions in malathion treated groups compared to control groups, in both larval and adult insects (Fig. 1B).

### Oxidative damage assay

The mean protein carbonyls amount recorded higher values in treated larvae and adults than in control, except for the highest treated group (0.02 mg/ml group) (Table 1). It was elevated about 27.7% in the 0.015 mg/ml MHL group and 44.1% in the 0.005 mg/ ml MHA, compared to the control groups. However, the protein carbonyls amount showed almost the same values as the control groups in both 0.02 mg/ml MHL and MHA groups.

**Table 1:** The macromolecules oxidative damage in form of amount of protein carbonyls (OD/ mg protein), and concentration of lipid peroxides (mM cumene hydroperoxide/ mg protein). The protein carbonyls and lipid peroxides concentrations were obtained from gut homogenates of 5th instar larvae and males of *Hermetia illucens*, which their food containers were exposure to different concentration of malathion (0, 0.005, 0.01, 0.015, 0.02 mg/ml).

Malathion concentration (mg/ml)	Protein carbonyls amount (OD/mg protein)		Lipid peroxidation concentration (mM cumene hydroperoxide/ mg protein)	
	Larvae (MHL)	Adult (MHA)	Larvae (MHL)	Adult (MHA)
0	36.56 ± 2.4a	43.8 ± 0.1a	11.47 ± 0.3	34.4 ± 0.5a
0.005	38.52 ± 1.6ab	62.7 ± 2.5	34.11 ± 1.4ab	38.4 ± 1.9ac
0.01	42.64 ± 0.8bc	48.3 ± 1.6ab	31.07 ± 0.8a	22.35 ± 0.4b
0.015	46.56 ± 0.3c	48.9 ± 0.3b	35.68 ± 1.5b	39.21 ± 1.1c
0.02	36.17 ± 1.9a	43.6 ± 1.2a	22.74 ± 1.4	26.1 ± 1.55b

The data were expressed as mean ± SE.

Mean values marked with the different lowercase letters were significantly different among malathion concentration assessment (ANOVA test *Tukey's-b*, Post Hoc test, *p* < 0.05).

The lipid peroxidation concentration reached its highest value, for both MHL and MHA, at 0.015 mg/ml malathion concentration. It was increased by 3.11-fold, and 1.14-fold, respectively, compared to the control values (Table 1). Meanwhile, the macromolecules oxidative damage, in form of Lipid peroxidation concentration, was decreased in MHL and MHA 0.02 mg/ ml treated groups, where it recorded lower value than control group in MHA.

### Enzymatic antioxidant response

**Figure 2:**
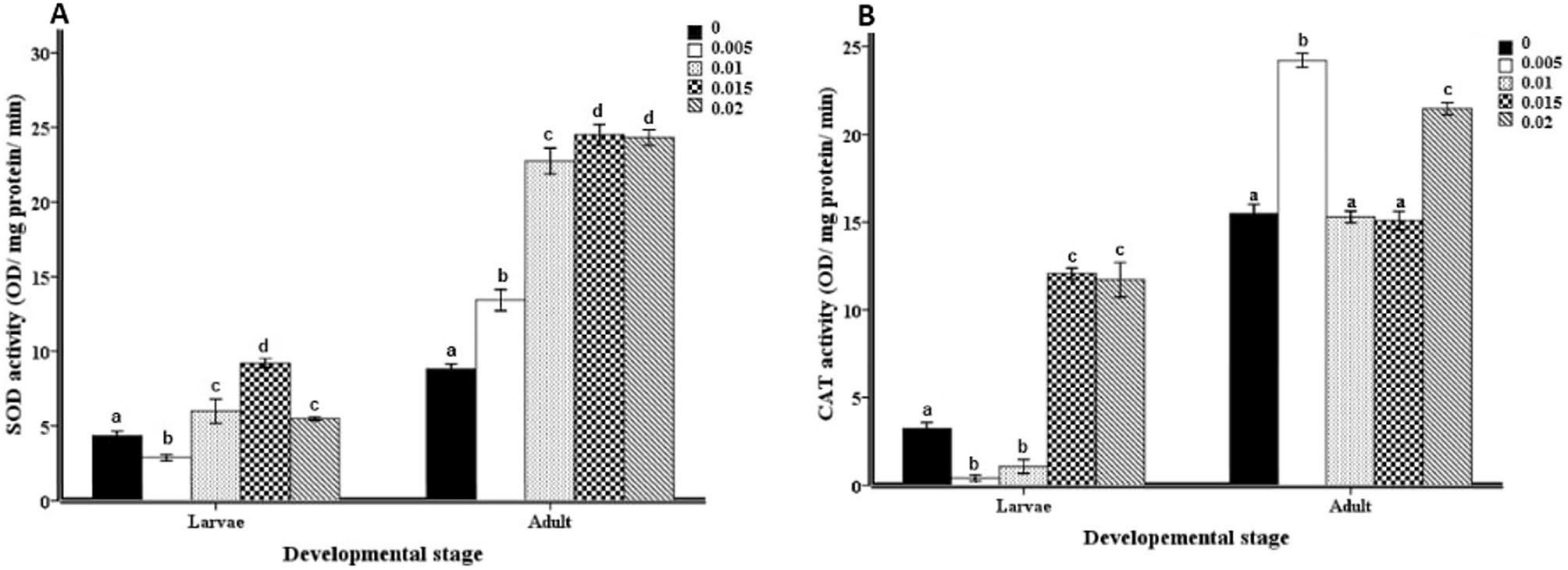
Activity of antioxidant enzymes in form of: (**A**) superoxide dismutase (SOD), (**B**) catalase (CAT), and (**C**) polyphenol oxidase (PPO) (OD/ mg protein/ min). The enzymes activity was expressed as mean ± SE values. The enzymatic antioxidant activity obtained from gut homogenates of 5th instar larvae and males of *Hermetia illucens* which their food containers were exposure different concentration of malathion (0, 0.005, 0.01, 0.015, 0.02 mg/ml). Mean values marked with the different lowercase letters were significantly different among malathion concentration assessment (ANOVA test *Tukey's-b*, Post Hoc test, *p* < 0.05).

In the control groups of *H. illucens*, the antioxidant enzymatic response, expressed as SOD and CAT, was significantly lower in the larval stage than in adult stage (Fig. 2A and B). Both SOD and CAT antioxidant activity were significantly (*p* < 0.05) higher in MHL and MHA 0.02 mg/ml malathion groups than the control groups (Fig. 2A and B). Yet, there was a significant (*p* < 0.05) decrease in PPO activity at 0.02 mg/ml malathion concentration than control groups at larval and adult stages (Fig. 2C).

### Non-enzymatic antioxidant response

**Figure 3:**
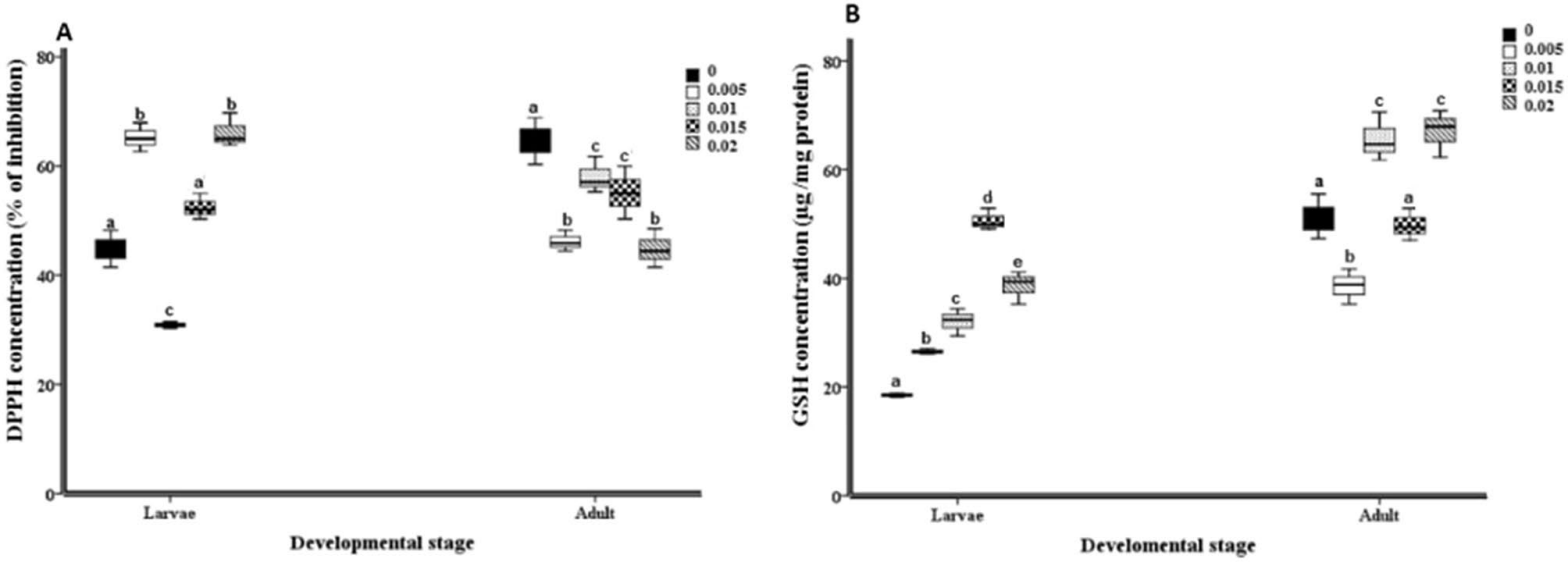
Activity of non-enzymatic response in form of the concentration of anti-radical 2,2-diphenyl-1-picrylhydrazyl (DPPH) (percentage of inhibition) (**A**), and antioxidant glutathione reduced (GSH) (µg/ mg protein) (**B**). The non-enzymatic antioxidants concentration was expressed, in a box-plot graph, as median, min., max., P25, and p75 values. The non- enzymatic antioxidant concentration obtained from gut homogenates of 5th instar larvae and males of *Hermetia illucens*, which their food containers were exposure different concentration of malathion (0, 0.005, 0.01, 0.015, 0.02 mg/ml). Mean values marked with the different lowercase letters were significantly different among malathion concentration assessment (ANOVA test *Tukey's-b*, Post Hoc test, *p* < 0.05).

The values of non-enzymatic antioxidant responses were represented in Fig. 3A and B. While DPPH recorded significantly higher value at 0.005 and 0.02 mg/ml malathion treated groups than the control group. In addition to the DPPH concentration of the control adult was significantly higher than all the treated adult groups (Fig. 3A). On the contrary, GSH concentration elevated significantly in all the treated larval groups, and in 0.01, and 0.02 mg/ml malathion adult groups compared to the control groups of *H. illucens* (Fig. 3B).

### Relation and interaction assessment

Pearson′s correlation analysis between malathion concentration and oxidative stress assays (ROS concentration, macromolecules damage, enzymatic and non-enzymatic antioxidant assays), revealed the most significant relationship in both larvae and adult stages (Table 2). In larval stage, there was a correlation at *p* < 0.001 between malathion concentration and H_2_O_2_, PPO and GSH. Also, the strong correlation at *p* < 0.05 level was observed between malathion concentration (0–0.02 mg/ml) and SOD or CAT. Yet, there was no significant correlation between malathion concentration at adult stage, except for SOD at *p* < 0.001, and PPO, DPPH and GSH at *p* < 0.05 level (Table 2). However, the GEE interaction analysis between malathion concentration, developmental stage, interaction and intercept showed a significant influence between these factors in all oxidative stress parameters except in case of developmental stage effect on the levels of DPPH (Table 3).

**Table 2:** Pearson′s correlation coefficient to detect the effect of malathion different concentration (0, 0.005, 0.01, 0.015, and 0.02 mg/ml) on reactive oxygen species concentration (ROS), (in form of hydrogen peroxide (H_2_O_2_), superoxide anion radical (O_2_^•-^)), macromolecules damage (in form of protein carbonyls amount and lipid peroxides concentration), enzymatic antioxidant response (inform of superoxide dismutase (SOD), catalase (CAT), and polyphenol oxidase (PPO)), and non-enzymatic antioxidant response (inform of anti-radical 2,2-diphenyl-1-picrylhydrazyl (DPPH) and antioxidant glutathione reduced (GSH)) in the gut homogenates of 5th instar larvae and males of *Hermetia illucens*.

Category	Assessment	stage	r	Equation	R^2^	Type
ROS	H_2_O_2_	Larvae	0.91	Y = 1234.6 X	-0.8	Linear equation for prediction
		Adult	− 0.17	Y = 3056.2 X	− 15.1	
	O_2_^•-^	Larvae	− 0.06	Y = 1152.8 X	− 75.3	
		Adult	0.36	Y = 1398.2 X	− 118.9	
Macromolecules damage	Protein carbonyls	Larvae	0.22	Y = 2721.4 X	− 23.4	
		Adult	− 0.27	Y = 3204.7 X	− 17.1	
	Lipid peroxides	Larvae	0.37	Y = 1962.2 X	− 1.8	
		Adult	− 0.31	Y = 2036.6 X	− 8.3	
Enzymatic antioxidants	SOD	Larvae	0.57*	Y = 429.2 X	− 0.7	
		Adult	0.92**	Y = 1531.4 X	− 0.01	
	CAT	Larvae	0.58*	Y = 407.3 X	0.3	
		Adult	0.11	Y = 1239.9 X	− 7.2	
	PPO	Larvae	− 0.91**	Y = 996.1 X	− 3.5	
		Adult	− 0.52*	Y = 1332.2 X	− 11.1	
Non-enzymatic antioxidants	DPPH	Larvae	0.35	Y = 3739.8 X	− 2.9	
		Adult	− 0.51*	Y = 3372.9 X	− 16.3	
	GSH	Larvae	0.82**	Y = 2648.3 X	− 0.4	
		Adult	0.54*	Y = 3917.6 X	− 5.3	

* significant at *p* < *0.05*; ** significant at *p* < *0.001*.

**Table 3:** Generalized Estimating Equation to analyze the malathion concentration (0, 0.005, 0.01, 0.015, and 0.002 mg/ml), insect developmental stage (5th larval instar and adult males), combined effect of malathion concentration with insect developmental stage and finally intercept on reactive oxygen species concentration (ROS), (inform of hydrogen peroxide (H_2_O_2_), superoxide anion radical (O_2_^•-^)), macromolecules damage (inform of protein carbonyls amount and lipid peroxides concentration), enzymatic antioxidant response (inform of superoxide dismutase (SOD), catalase (CAT), and polyphenol oxidase (PPO)), and finally non-enzymatic antioxidant response (inform of anti-radical 2,2-diphenyl-1-picrylhydrazyl (DPPH) and antioxidant glutathione reduced (GSH)) in the gut homogenates of 5^th^ instar larvae and adult males of *Hermetia illucens.*

Category	Source	*Chi-square*	*df*	*p-value*	QIC*
**Concentration effect**					
ROS	H_2_O_2_	629.9	4	< 0.000	63.5
	O_2_^•-^	95.3	4	< 0.000	23.5
Macromolecules damage	Protein carbonyls	128.8	4	< 0.000	167.1
	Lipid peroxides	434.0	4	< 0.000	117.5
Enzymatic antioxidants	SOD	5535.3	4	< 0.000	24.4
	CAT	1001.3	4	< 0.000	132.2
	PPO	2401.3	4	< 0.000	30.3
Non-enzymatic antioxidants	DPPH	122.67	4	< 0.000	500.3
	GSH	405.6	4	< 0.000	210.3
**Developmental stage effect**					
ROS	H_2_O_2_	6471.9	1	< 0.000	63.5
	O_2_^•-^	564.1	1	< 0.000	23.5
Macromolecules damage	Protein carbonyls	134.9	1	< 0.000	167.1
	Lipid peroxides	59.9	1	< 0.000	117.5
Enzymatic antioxidants	SOD	8777	1	< 0.000	24.4
	CAT	393.8	1	< 0.000	132.2
	PPO	20.1	1	< 0.000	30.3
Non-enzymatic antioxidants	DPPH	0.81	1	> 0.05	500.3
	GSH	530.1	1	< 0.000	210.3
**Concentration × developmental stage effect**					
ROS	H_2_O_2_	5157.2	4	< 0.000	63.5
	O_2_^•-^	92.6	4	< 0.000	23.5
Macromolecules damage	Protein carbonyls	84.8	4	< 0.000	167.1
	Lipid peroxides	865.6	4	< 0.000	117.5
Enzymatic antioxidants	SOD	2871.2	4	< 0.000	24.4
	CAT	1940.3	4	< 0.000	132.2
	PPO	1703.5	4	< 0.000	30.3
Non-enzymatic antioxidants	DPPH	522.3	4	< 0.000	500.3
	GSH	254.5	4	< 0.000	210.3
**Intercept effect**					
ROS	H_2_O_2_	17,370.3	1	< 0.000	63.5
	O_2_^•-^	88,702.9	1	< 0.000	23.5
Macromolecules damage	Protein carbonyls	12,279.8	1	< 0.000	167.1
	Lipid peroxides	8068.8	1	< 0.000	117.5
Enzymatic antioxidants	SOD	29,937.1	1	< 0.000	24.4
	CAT	1025.1	1	< 0.000	132.2
	PPO	38,556.1	1	< 0.000	30.3
Non-enzymatic antioxidants	DPPH	5268.4	1	< 0.000	500.3
	GSH	9106.6	1	< 0.000	210.3

* Quasi Like hood under Independence Model Criterion.

**Figure 4:**
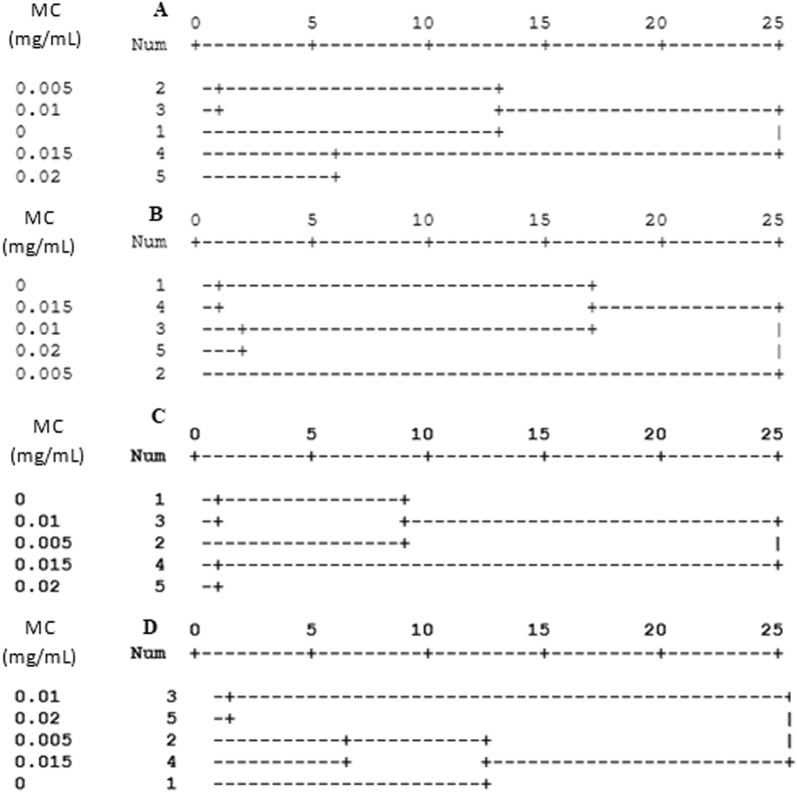
Dendrogram of the cluster analysis (using Ward′s Method) applied for oxidative stress assessment (in form of cluster the reactive oxygen species concentration (ROS), and macromolecules damage) in the gut homogenate of 5th larval instar (**A**), and male adult stage (**B**) of *Hermetia illucens.* The cluster analysis which applied for antioxidant response system (inform of enzymatic antioxidant response and non-enzymatic antioxidant response) in the gut homogenate of 5th larval instar (**C**), and male adult stage (**D**) of *Hermetia illucens*, which their food containers were exposure to different concentration of malathion (0, 0.005, 0.01, 0.015, 0.02 mg/mL).

The Dendrogram of the cluster analysis, using Ward′s Method, revealed slightly dissimilar patterns for control insect groups and 0.02 mg/ml malathion concentration. The clustering oxidative stress assessment and antioxidant response of both larval and adult stages were shown in Fig. 4a–d. The level of oxidative stress assessment was highly similar in larval stage at 0, 0.005, and 0.01 mg/ml malathion concentration (Fig. 4a) however, the cluster of ROS and macromolecules damage occurred in adult stage at 0, 0.005, 0.01, and 0.015 mg/ml malathion (Fig. 4b). The 0, 0.005, and 0.01 mg/ml malathion concentration created a separate cluster in antioxidant response system of larval *H. illucens* (Fig. 4c), though, in adult stage 0, 0.005, and 0.015 mg/ml malathion formed a separate cluster in antioxidant enzymatic and non-enzymatic response in gut homogenates of *H. illucens* (Fig. 4d).

**Figure 5:**
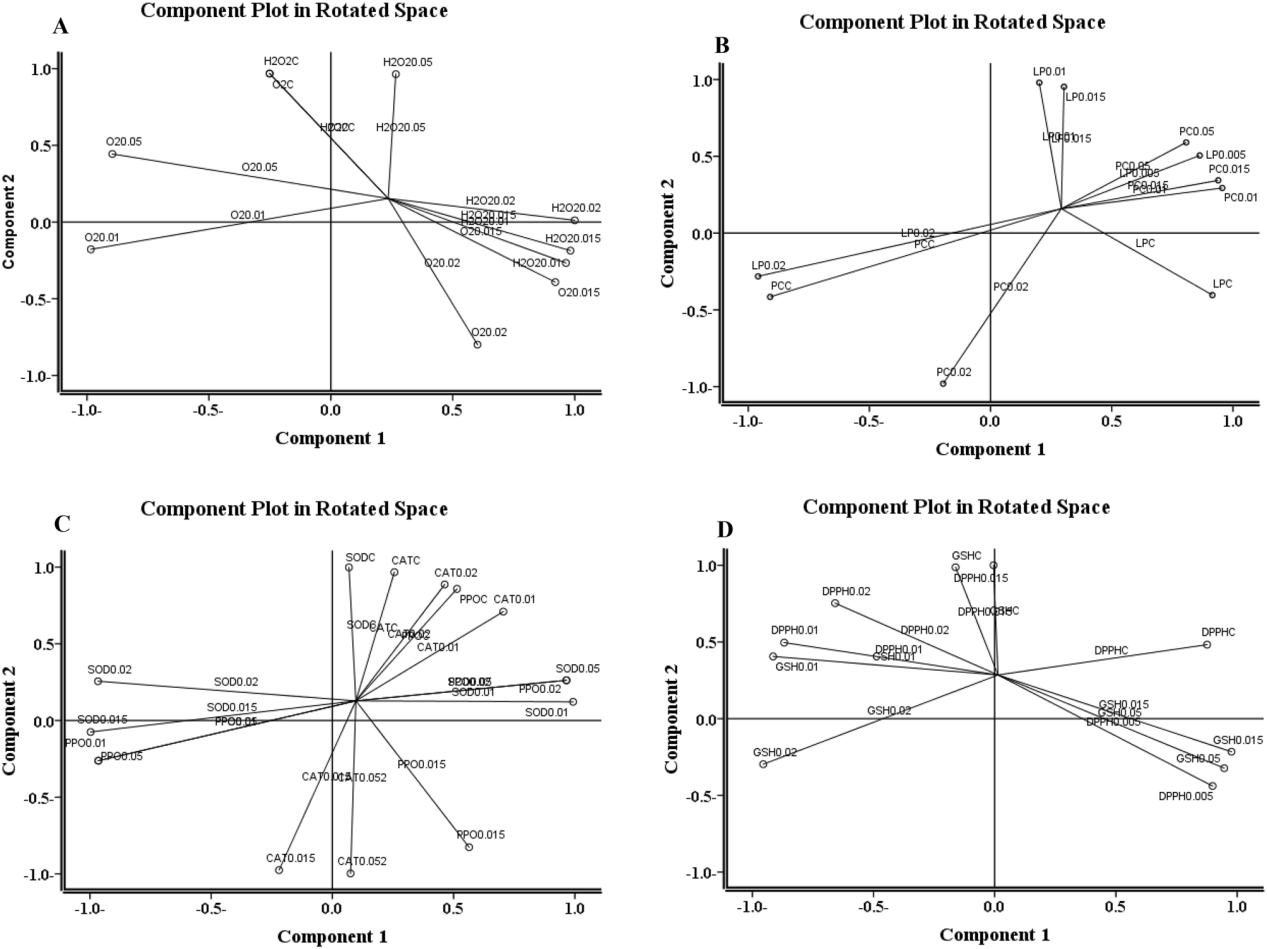
Environmental computing of different malathion concentration (0, 0.005, 0.01, 0.015, and 0.02 mg/ml) and developmental stage effect (5th larval instar, and adult male) in form of principal component analysis (PCA). The PCA was analyzed the two different component, which applied for reactive oxygen species concentration (ROS), (inform of hydrogen peroxide (H_2_O_2_), superoxide anion radical (O_2_^•-^)) (**A**), macromolecules damage (inform of protein carbonyls amount and lipid peroxides concentration) (B), enzymatic antioxidant response (inform of superoxide dismutase (SOD), catalase (CAT), and polyphenol oxidase (PPO)) (**C**), and finally non-enzymatic antioxidant response (inform of anti-radical 2,2-diphenyl-1-picrylhydrazyl (DPPH) and antioxidant glutathione reduced (GSH)) (**D**) in the gut homogenates of *Hermetia illucens.*

Environmental computing of the effect of different malathion concentration (0, 0.005, 0.01, 0.015, and 0.02 mg/ml) and developmental stage (5th larval instar and adult male) were shown in Fig. 5a–d. These effects were assessed in the form of principal component analysis (PCA). The PCA was done through variance co-variance matrix analysis with two different components. Also, the eigen value tended to be dependent on 2 variables which were classified into first component and second component. The 1st component is different malathion concentration and the 2nd one is developmental stage*.* The normalization rotation method revealed that the O_2_^•-^ production rate, in groups treated with malathion concentration from 0 to 0.01 mg/ml, had a great ROS levels in larval and adult stages (Fig. 5a). Meanwhile, the macromolecules damage, in the form of protein carbonyls and lipid peroxide, was detected at concentration 0 or 0.02 mg/ ml malathion (Fig. 5b). The variance–covariance analysis showed a high variability of enzymatic antioxidant system along malathion concentration (Fig. 5c). However, the first component of PCA tended to be localized centric and reflected between non-enzymatic response and malathion concentration (Fig. 5d).

## Discussion

Many studies focused on the role of *H. illucens* in organic waste recycling process, and the insect’s valuable products, such as chitin, lipids, and proteins, which could be used on various industrial applications^25–32^. The modern scientific researches focused on using BSF larvae, that fed on organic waste, as animal feed. These wastes might contain different kinds and concentrations of pesticides which affect both the insect bioreactor and the feeding animals. The new stress problems such as the pesticides application or the mechanisms beyond the accumulation process were considered as one of the most interesting research points which focused on the phenomena of hormesis, adaptation, and mitigation^33^. Moreover, the lethargic effect of humans on the environmental components led to a dangerous and even lethal, backlash from ecosystem especially living organisms^34^. Several of the previous studies approved the assessment of oxidative stress in the biomonitoring of environmental pollution^35–37^. Exposure to the pesticides may raise the oxidative stress status directly by increasing the reactive oxygen species (ROS) over generation rate of the non-enzymatic and enzymatic antioxidants in the cells^36,38^. Maintaining the normal levels of oxidants in the cells is of utmost priority to avoid the negative actions of oxidative stress products, such as protein carbonyls and lipid peroxides levels on the living organisms’ health^36,39^. Organophosphates could initiate ROS production and oxidation products in the cells^40,41^. malathion may indirectly increase the production of ROS inside the cells through disrupting the respiratory metabolism^42^, It also contains the P-S bond (“thion”) that may convert to P-O bond (“oxon”), by the action of a microsomal system of enzymes named mixed-function oxidases (MFO), and cytochrome P450 (CYP450)^43^. The oxon compounds are highly toxic and can initiate oxidative damage to the living cells. malathion has a toxic effect on pupa, male and female of the peach fruit fly, *Bactrocera zonata*, with a higher resistance ratio in the field population than in laboratory insect population^44^. In the current study, a slight increase in the H_2_O_2_ and O_2_^•-^ concentration was observed in the MHA than MHL by using all malathion concentration course (0–0.02 mg/ml) (Fig. 1). This explained the oxidants accumulation possibility^38^ or the antioxidants levels’ depletion in the adult stage than in the immature stage^36^. However, the H_2_O_2_ concentration in 120th generation old of *Spodoptera exigua* didn't change after the exposure to 44 μg/g of dry weight Cd^45^. The efficiency of the elimination process of oxidants and oxidative products may be impaired in the stressful conditions. This phenomenon occurred in this study where, the ROS, as O_2_^•-^ production rate, of the MHA increased than MHL (Fig. 1b). Also, *H. illucens* can normalize the concentration of H_2_O_2_ and O_2_^•-^ especially in MHA. However, when the MHL was exposed to high concentration of malathion (0.02 mg/ml), the concentration of H_2_O_2_ increased significantly than control values (Fig. 1a). Similarly, the toxicity of OP compounds induced some oxidative stresses, like elevation in the protein carbonyls amount or lipid peroxides level in some living organisms^46,47^, and caused physiological and pathological changes in tissues^48^. The LC_50_ value of methidathion pesticide could affect the malondialdehyde level and antioxidant enzyme activities in the gut tissues of *Lymantria dispar* (Lepidoptera) larvae^49^. Similarly, our results recorded the highest concentration of protein carbonyls amount at 0.015 mg/ml malathion in the MHL, and the highest lipid peroxides concentration in MHL, and MHA at the same malathion concentration (Table 1). Yet, there was no significant difference between the highest concentration of malathion (0.02 mg/ml) and control (0 mg/ml) in both MHL and MHA (Table 1). This may be due to the action of enzymatic and non-enzymatic response^36^. The current study detected fluctuations in the concentration of protein carbonyls throughout the malathion concentration-course (Table 1), and this may reflect on the fluctuating homeostatic mechanism balance between protein degradation and production of protein carbonyls^36,50^. Lipid peroxidation can disrupt the membrane of the polyunsaturated phospholipids bilayer structure and function^51^. Also, products of lipid peroxidation are capable of disrupting conformations of many cellular proteins, including enzymes, by forming cross links with these proteins, inactivating their functions^52^. Lipid peroxidation is considered as a chain reaction; it produces lipid radical, lipid peroxyl radical, and then lipid hydroperoxide. This reaction can be stopped by termination reactions, such as the recombination of lipid peroxyl radicals and by a reaction with glutathione catalyzed by peroxidase^53^. Therefore, the pattern of fluctuation of lipid peroxides in *H. illucens*, formed post treatment with different concentration of Malathion, may be due to unbalanced levels of lipid peroxides production and their repairing mechanisms that may include antioxidant enzymes**.**

Generally cited that, malathion is considered as neurotoxic component that inhibits the neuronal cholinesterase enzyme activity. Also, the peroxidative effects of OP were studied on the activities of antioxidant enzymes, and on lipid peroxidation *in-vitro*^54^, and *in-vivo* studies^55^. malathion treatment resulted in the elevation of lipid peroxidation concentration which was considered as an indicator of oxidative stress induction^56^. The results showed that the malathion applications led to elevation in the activities of the key antioxidant enzymes, SOD and CAT, over the constitutive levels except for different cases of malathion concentration (Fig. 2a and b). The observed elevation seemed to occur in concomitance with the oxidative damages of the macromolecules; and may be in response to the formation of ROS as a consequence to the treated stressor^36^. SOD and CAT have a primary role in the oxidative stress defense through ROS elimination^53^. However, the significant depletion of PPO activity, which catalyzes the oxidation of phenolic compounds to quinones, occurred in both MHL and MHA treatments along concentration course of malathion (Fig. 2c). malathion treatment could acidify the medium and inhibit the PPO activity^57^. The DPPH results of MHL showed a significant increase in the 0.005 and 0.015 mg/ml malathion concentration treatment, compared to control values. Similarly, the malathion applications caused a serious risk to *Saccharomyces cerevisiae* (fungus). This *in-vitro* study showed that a flavonoid compound called naringin can inhibit some enzymes and can detoxify the DPPH radical^57^. Glutathione (GSH) acts as a redox factor to balance the redox state of the cell^58^. Reduced GSH is a chief cellular thiol element in the antioxidative system. Additionally, GSH and DPPH have a significant ROS scavenging role. The chemical stressors can increase the glutathione concentration in animals^59^. However, the one generation of *Spodoptera exigua* (Lepidoptera) which was exposed to Cd, didn't show elevation in the GSH concentration^60–62^. Our results showed that, the increase in GSH concentration was significant in both MHL and MHA, especially, at 0.015, 0.01 and 0.02 mg/ml malathion concentration, respectively (Fig. 3b).

The interaction analysis, obtained from the computation of generalized estimating equation (GEE), revealed that the different concentration of malathion (0, 0.005, 0.01, 0.015, 0.02 mg/ml), the different developmental stage (MHL and MHA), and the interaction of these terms significantly influenced the physiological endpoints we measured (H_2_O_2_,O_2_^•-^, protein carbonyls, lipid peroxides, SOD, CAT, PPO, DPPH, and GSH) (*p* value < 0.05), except for DPPH in the developmental stage effect (*p* value > 0.05) (Table 3).

It has been concluded that the malathion exposure of insect food container can induce oxidative stress in the larval and adult male stages of *H. illucens*. The levels of ROS, macromolecules damage, enzymatic and non-enzymatic response in BSF to different malathion concentration may be used as a possible mechanism of malathion toxicity. The biochemical analysis of insects could be used as a novel strategy for assess the risk of pesticides accumulation on food container of recyclers of organic waste especially insects. Briefly, the tested hypothesis in this research has proved the ability of using oxidative stress parameters as bioindicator of malathion impact on the organic waste recycler, BSF. Meanwhile, malathion has uniform prooxidant properties in BSF, unlike the other phenolic compounds related to insecticides. The upcoming work will further investigate the fate of malathion in BSF, will answer a question about the role of the insect in reducing the toxicity and severity of malathion, and will shed more light on its role in bioremediation.

## Materials and methods

### Insects rearing and Malathion application

A colony of *H. illucens* was supplied from Al Qalyobia governorate and was reared under laboratory conditions for several generations, in the Department of Entomology, Faculty of Science, Cairo University. The experiments were made at summertime 2020, where the rearing conditions were (14:10 L:D; 34˚ ± 2; 60% RH) for adults and (0:24 L:D; 34˚ ± 2; 75% RH) for larvae. The insects were kept in mesh cages 30*30*40 cm^3^ for adults (100 adults/ cage), and 20*20*10 cm^3^ for larvae (200 larvae/ cage). Larvae were supplied daily with kitchen waste, 1:1 vegetable: fruit waste, (1000 larvae/ one kg kitchen waste) from household located at Giza Governorate, while adults were hydrated with water and sugar^24^.

The malathion application was performed by immersing the insect food container with different malathion concentrations (0, 0.005, 0.01, 0.015, or 0.02 mg/ml) for 24 h. Simultaneously, control insects were treated with immersing food container with distilled water. The levels of oxidative stress parameters of control insects were taken 100% levels. The range of low-level insecticide contamination of malathion were applied. Insects were divided into 2 groups: 5th larval instar, and male adult. The adult experiment specimens were taken into account the male’s not females insects in order to avoid the compounding effects of ovarian development in the female insects. The sex of adult specimens was differentiating according to the insect morphological characters; where the males are characterized by a rounded genital apparatus. Each group was divided into 5 sub-groups of 250 individuals, which were exposed to malathion (0, 0.005, 0.01, 0.015, or 0.02 mg/ml) for 24 h post application. For each sub-group, 50 insects were dissected, after 24 h’ malathion application, to isolate gut tissues.

About 7.5 gm gut tissues, of each experimental sub-group, were homogenate in 7.5 ml ice-cold phosphate buffer (50 mM; pH 7.0 contained, 1 ml of 0.1% Triton X-100, 1 ml of 0.05 mM CaCl_2_); and were centrifuged at 2000 × *g* for 10 min at 4 °C. The clear sample were stored at − 20 °C until use for further analysis. Each experiment was replicated three times.

### The concentration of ROS

The concentration of H_2_O_2_ was determined spectrophotometrically according to the method of Junglee et al.^63^. Briefly, using one step extraction-colorimetric procedure in which, homogenization step using PBS, pH = 7.0 mixed with 0.25 ml Trichloroacetic acid (TCA) (0.1% (w:v)), 0.5 ml KI (1 M), then the 1 ml samples were centrifuged at 12,000 × g for 15 min at 4 °C, and finally, the absorbance was measured at 240 nm. For the superoxide anion radical (O_2_^•-^) production rate of samples was determined using colorimetric analysis according to the method of Chen and Li^64^. The reaction mixture contains 0.25 mL epinephrine (1 mM), 0.25 mL NADPH (1 mM), 0.5 mL sodium phosphate buffer (PBS) (50 mM; pH 7.0), and 1 mL of the samples. The level of superoxide anion radical was determined by the rate of conversion of epinephrine to adrenochrome with 1 mM NADPH as substrate. The absorbance difference (A_485_–A_575_) was recorded.

### Oxidative damage assay

Protein carbonyls amount assay was performed according to procedure from Levine et al.^65^. After homogenization and centrifugation steps, a mixture of 800 µL sample, and 200 µl 2, 4-dinitrophenyl hydrazine (DNPH) (10 mM) was incubated for 30 min at room temperature, then precipitated with 1 ml TCA (1%). The pellet was washed four times with 1 ml absolute ethanol/ethyl acetate (1:1) mixture and dissolved in 1 mL of PBS (50 mM; pH 7.0), before being measured at 366 nm.

The lipid peroxides concentration was measured according to Hermes-Lima et al.^66^. After homogenization and centrifugation step, a mixture of 200 µl sample, 400 µl FeSO_4_ (1 mM), 200 µl H_2_SO_4_ (0.25 M)_,_ and 200 µl xylenol orange (1 mM) were added, then absorbance measured at 580 nm. The mixture was incubated in the dark for 3 h at room temperature, and finally the absorbance re-measured at 580 nm after adding of 10 µl cumene hydroperoxides (0.05 mM) (as an internal standard). The change in absorbance due to addition of internal standard was calculated.

### Antioxidant enzymatic response

SOD activity was measured based on the procedure described by Misra and Fridovich^67^. The reaction mixture was as follows: 0.4 ml of a sodium carbonate buffer (200 mM; pH 10.0), 35 µl of EDTA (10 mM), 87 µl of the sample and 0.5 ml of freshly prepared epinephrine (15 mM). The absorbance was measured at 480 nm.

The activity of CAT was assessed in compliance with the method of Aebi^68^. The reaction mixture contained 0.9 ml of a potassium phosphate buffer (50 mM, pH 7.0), 60 µl of the sample and 40 µl of freshly prepared H_2_O_2_ (10 mM). The change in absorbance was measured at 240 nm over a period of 0.5 min. The method of Kumar and Khan^69^ was used to assess the PPO activity in a reaction mixture containing 0.9 ml of a potassium phosphate buffer (50 mM, pH 7.0), 0.25 ml of 0.1 M catechol and 0.25 mL sample. The reaction formed purpurogallin which was measured at 495 nm.

### Antioxidant non-enzymatic response

DPPH antioxidant activity was determined according to Blois^70^, by adding 0.5 ml DPPH (o.5 M) to 0.5 ml sample and incubated for 20 min before measuring absorbance at 525 nm. DPPH assay was based on the scavenging capability measurement. The nitrogen atom contains an odd electron which is reduced by delivering a hydrogen atom from antioxidants to hydrazine. The procedure of Allen et al.^71^ was adapted for determining GSH concentration. Briefly, the reaction mixture, containing 150 μl sample, 800 μl PBS (50 mM; pH 8.0), and 50 μl 5, 5´-Diothio bis-2-nitrobenzoic acid (2 mM), then incubated at 25 °C for 20 min. The absorbance of the reaction mixture was 412 nm. The GSH content was determined from a GSH standard curve. The total protein concentration of samples was determined spectrophotometrically according to the method of Bradford^72^. Briefly, 0.9 mL of the Coomassie brilliant blue (0.5 mM) were mixed with 0.1 mL sample and incubated at room temperature for 2 min. The OD of the protein sample was measured at 595 nm.

### Statistical analysis, relation, and interaction assessment

Statistical analysis was performed using IBM SPSS Statistics for Windows (Version 17.0. Armonk, NY: IBM Corp.). A parametric test was carried out using ANOVA test *Tukey's-b*, Post Hoc test for assessment the malathion concentration effect and T-test, for insect developmental stage. Correlations between malathion concentration and the experimental assays of oxidative stress parameters, including, ROS, macromolecules damage, enzymatic antioxidant response and non-enzymatic antioxidant response, were performed based on Pearson’s regression analysis using linear regression models. Hierarchical Cluster Analysis (HACA) based on agglomerative statistics using Ward′s Method was calculated for oxidative stress parameters. The goal of HACA is to find possible clusters or groups among the observational units, based on level of similarities and differences^12^. Generalized Estimating Equation (GEE) was used to examine the effect of malathion concentration, insect developmental stage, combined effect of concentration and developmental stage, and finally intercept on the parameters of oxidative stress. The principal component analysis (PCA) was performed the possible assessment of different malathion concentration and developmental stage on the oxidative stress parameters of BSF.

### Ethical approval and consent to participate

This article does not contain any studies with human participants or animals that require ethical approval.

## Data Availability

The datasets used and/or analyzed during the current study are available from the corresponding author on reasonable request.

## Abbreviations

ROS: Reactive oxygen species
H_2_O_2_: Hydrogen peroxide
O_2_^•-^: Superoxide anion radicals
OP: Organophosphate pesticides
DPPH: α, α-Diphenyl-β-picrylhydrazyl
GSH: Reduced glutathione
SOD: Superoxide dismutase
CAT: Catalase
Px: Peroxidase
PPO: Polyphenol oxidase
APOx: Ascorbate peroxidase
AChE: Acetylcholine esterase
MHL: The midgut homogenates of 5th larval instar of *Hermetia illucens*
MHA: The midgut homogenates of male adult of *Hermetia illucens*
TCA: Trichloroacetic acid
DNPH: 2, 4-Dinitrophenyl hydrazine
PBS: Sodium phosphate buffer
HACA: Hierarchical Cluster Analysis
PCA: Principle component analysis
GEE: Generalized Estimating Equation
